# Constitution and By-laws of the Veterinary Medical Association of New York County

**Published:** 1894-09

**Authors:** 


### CONSTITUTION AND BY-LAWS OF THE VETERINARY MEDICAL ASSOCIATION OF NEW YORK COUNTY.

---

### *CONSTITUTION.*

ARTICLE I.—This Association shall be known as the Veterinary Medical Association of New York County.

ART. II.—The objects of the Association are for the mutual improvement of its members, and the advancement and welfare of the veterinary profession.

ART. III.—The officers shall be a President, a Vice-President, a Secretary, a Treasurer, and a committee of five (5) members, who shall be known as the Board of Censors.

### *BY-LAWS.*

ARTICLE I.—The order of business shall be as follows:

1.—Roll call.
2.—Reading of minutes of previous meeting.
3.—Report of Comitia Minora.
4.—Papers and discussion.
5.—Reports of all other committees.
6.—Admission of new members.
7.—Application for membership.
8.—Election of officers.
9.—New business.

ART. II.—The President shall conduct all meetings in accordance with parliamentary usage.

The President shall appoint all committees, unless otherwise ordered by special vote of the meeting. He shall appoint essayists to read papers for the regular meeting next succeeding.

ART. III.—The Vice-President, in the absence of or at the request of the President, shall perform the duties of his (the President's) office.

ART. IV.—The Secretary shall perform the usual duties pertaining to the office. The Secretary shall be exempt from payment of annual dues.

The Secretary shall be allowed an annual salary of fifty (50) dollars.

Art. V.—The Treasurer shall receive all moneys of the Association, and pay all bills duly audited and found correct by the President and Secretary.

The Treasurer shall present a detailed statement of the business of the Association at the Annual Meeting, or at any other time by special resolution.

Art. VI.—A Board of Censors shall be appointed by the President at each annual meeting. It shall be the duty of the Board of Censors to examine the credentials and vouchers of all applicants for membership.

They shall report in writing the result of their examination to the President of the Association.

The Board of Censors shall be invested with power to hear or determine upon complaints filed before them in writing, relative to the improper or immoral conduct of any member, and shall, if thought advisable, report upon such complaint to the Association at the next regular meeting, the offending member being duly notified of such complaint and allowed the privilege of defence ; and such member, if deemed guilty by a vote of two-thirds of the members present, shall cease to be a member of the Association.

Art. VII.—Members to consist of

1. Charter Members.—All those practitioners of veterinary medicine who assist in the organization of the above Association, and conform to the regulations formulated by a two-thirds vote of those in attendance at the meeting of organization.

2. Future Members.—Applicants for membership shall submit their names upon one of the Association's application blanks, duly vouched for by two members of the Association.

They shall be graduates of a regularly organized and recognized veterinary school, which has a curriculum of at least three years, of six months each, specially devoted to the study of veterinary science, and whose corps of instructors shall contain at least four veterinarians. Second, they shall be of good moral character and of reputable business methods.

Art. VIII.—A member elect shall, within one month, pay to the Treasurer his initiation fee and annual dues—whereon he shall sign the Constitution and By-Laws and receive his certificate of membership.

Art. IX.—The form of certificate to be as follows:

Heading: The Veterinary Medical Association of New York County.

This is to certify that the Veterinary Medical Association of

New York County, organized January 25, 1894, do hereby confer upon *John Doe* this, their certificate of membership, as required by the rules and by-laws of said Association.

*Censors*.................... *Pres*...................

*L. S.* .................... *Secy*...................

Art. X.—Honorary members may be proposed in writing by any three (3) members of the Association, stating the grounds upon which such application is made. Honorary members must be persons who have aided in the advancement of the veterinary profession.

Honorary members in debate shall have the same privileges as other members, but shall have no vote.

Not more than three (3) honorary members shall be elected annually.

Art. XI.—The initiation fee shall be five dollars ($5.00). The annual subscription shall be two dollars ($2.00).

Art. XII.—All members eighteen (18) months in arrears shall have their names stricken from the roll, after due notice, until such arrears are paid.

Art. XIII.—The Association shall meet upon the first Tuesday of each month; except during July, August and September.

Art. XIV.—The Judiciary Committee shall consist of five (5) members, appointed by the President. They shall keep a record of veterinary surgeons as to their registration, have charge of legislation and other legal matters coming before them, with any prosecutions of those not duly registered according to law.

Art. XV.—Any motion for alteration of By-laws must be presented in writing, and must be adopted by a two-thirds vote of the members present.

All members of the Association shall be notified at least two weeks previous to any action thereon.

### *CODE OF ETHICS.*

Article I.—No member shall assume a title to which he has not a just claim.

Art. II.—No member shall endeavor to build up a practice by undercharging his brother member.

Art. III.—No member shall speak disrespectfully of another, or in any way attempt to lessen his professional reputation, particularly for his individual advancement.

ART. IV.—In all cases of consultation it shall be the duty of the veterinary surgeon in attendance on the case to give the opinion of the consulting veterinary surgeon (whether favorable to his own or otherwise) to the owner of the patient in the presence of all three.

In case of the absence of the owner, the veterinarian consulted may, after giving his opinion to the attending veterinary surgeon, transmit it also in writing to the owner.

It shall be deemed a breach of this Code for a consulting veterinarian to revisit a patient without special invitation or agreement.

ART. V.—In advertising, the veterinary surgeon shall confine himself to his business address.

Advertising specific medicines, specific plans of treatment, advertising through the medium of posters, illuminated bills, newspapers or calendars, will not be countenanced by this Association.

ART. VI.—Any person who shall advertise or otherwise offer to the public any medicine the composition of which he refuses to disclose, or if he publicly proposes to cure disease by any such secret medicine, shall be deemed an unworthy member and be expelled from the Association.

ART. VII.—It shall be deemed a violation of the Code of Ethics for any member of this Association to contract with or through the officers of any live-stock insurance company, for the professional treatment of the members' stock so insured, but this rule shall not prevent any member from becoming the examiner of risks and to act in the capacity of an expert for the same.

ART. VIII.—Every member shall observe the Code of Medical Ethics adopted by this Association, and be answerable to the Board of Censors for any breach of the same.

ART. IX.—That all charges of a personal nature, or regarding breaches of professional etiquette, shall be made only in writing to the Secretary, by whom they shall be referred to the Board of Censors.

